# Intradialytic hypotension is an important risk factor for critical limb ischemia in patients on hemodialysis

**DOI:** 10.1186/s12882-019-1662-x

**Published:** 2019-12-19

**Authors:** Ryo Matsuura, Sumi Hidaka, Takayasu Ohtake, Yasuhiro Mochida, Kunihiro Ishioka, Kyoko Maesato, Machiko Oka, Hidekazu Moriya, Shuzo Kobayashi

**Affiliations:** 1 Kidney Disease and Transplant Center, Shonan Kamakura General Hospital, 1370-1, Okamoto, Kamakura, 247-8533 Japan; 2 Department of Nephrology and Endocrinology, The University of Tokyo, 7-3-1, Hongo, Bunkyo-ku, Tokyo, 113-8655 Japan

**Keywords:** Hemodialysis, Diabetes mellitus, Intradialytic hypotension, Critical limb ischemia, Risk factor

## Abstract

**Background:**

Critical limb ischemia (CLI) and intradialytic hypotension (IDH) are common complications in patients on hemodialysis (HD). However, limited data are available on whether IDH is related to CLI in these patients. The aim of this retrospective study was to evaluate whether IDH is a risk factor for CLI in HD patients.

**Methods:**

We examined the frequency of IDH in 147 patients who received HD between January 1 and June 30, 2012. Blood pressure was measured during HD every 30 min and IDH was defined as a ≥ 20 mmHg fall in systolic blood pressure compared to 30 min before and a nadir intradialytic systolic blood pressure < 90 mmHg. The primary study outcome was newly developed CLI requiring revascularization treatment or CLI-related death. We assessed the association of IDH with outcome using a multivariable subdistribution hazard model with adjustment for male, age, smoking and history of cardiovascular disease.

**Results:**

The median follow-up period was 24.5 months. Fifty patients (34%) had episodes of IDH in the study entry period. During follow-up, 14 patients received endovascular treatment and CLI-related death occurred in 1 patient. Factors associated with incident CLI in univariate analysis were age, smoking, diabetes mellitus, peripheral arterial disease, history of cardiovascular disease, and IDH. IDH was significantly associated with the outcome with the subdistribution hazard ratio of 3.13 [95% confidence interval, 1.05–9.37].

**Conclusions:**

IDH was an independent risk factor for incident CLI in patients on HD.

## Background

Critical limb ischemia (CLI), the most advanced form of peripheral arterial disease (PAD), is a serious complication of end-stage renal disease, along with cardiovascular disease (CVD) and cerebrovascular disease [1]. Moreover, CLI often complicates chronic infections and leads to limb amputations in these patients [2].

Revascularization treatment (endovascular therapy or bypass surgery) is recommended for patients with CLI [3]. However, the incidence of restenosis and re-occlusion after endovascular therapy is extremely high in CLI patients on hemodialysis (HD) when compared with those not on HD [4]. Amputation-free and overall survival rates are also extremely poor in HD patients with CLI ([4–6]). Therefore, it is important to predict the risks for incident CLI and provide multidisciplinary care for preventing this potentially lethal condition in patients on HD [3].

HD patients have combined risk factors (traditional, uremia-related, and dialysis-related risk factors) for atherosclerotic disease [7, 8]. Therefore, patients undergoing HD are at greater risk of PAD than the general population [9]. In our previous study, prevalence of PAD was very high, i.e., 41.4% of maintenance HD patients had PAD [10]. However, risk factors for incident CLI have not been fully elucidated in HD patients. Particularly, dialysis related risk factor for incident CLI still remains unknown in HD patients.

Hypotension during HD, known as intradialytic hypotension (IDH), is a serious complication in patients on HD. IDH causes abdominal discomfort, yawning, sighing, vomiting, and muscle cramps. IDH causes tissue hypoxia, leading to cardiovascular morbidity and mortality [11]. IDH has also been associated with poor 2-year survival outcome [12]. However, it has never been verified whether IDH is associated with incident CLI in HD patients. In this retrospective study, we investigated and elucidated the risk factors, including IDH, for incident CLI in patients on HD.

## Methods

### Study design

**Fig. 1:**
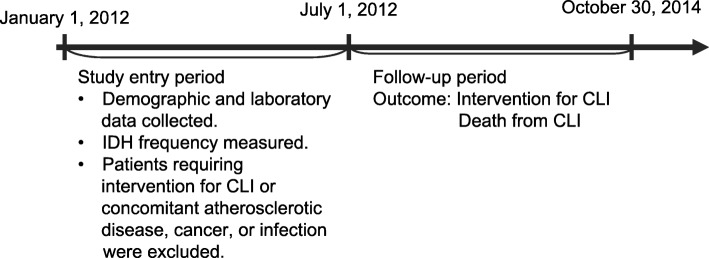
Outline of study protocol. Abbreviations: CLI, critical limb ischemia; IDH, intradialytic hypotension

We investigated the risk factors for incident CLI in patients on HD. The primary outcome was incident CLI requiring an endovascular and/or surgical procedure or CLI-related death. The design of this study is shown in Fig. 1. Patients with HD duration more than 3 months and received maintenance HD 3 times weekly on an outpatient basis from January 2012 to June 2012 (study entry period) at Shonan Kamakura General Hospital were eligible in this study. Exclusion criteria were 1) patients on hemodiafiltration, 2) patients who already had CLI at January 2012, 3) patients who underwent intervention for CLI during the 6-month entry period, and 4) patients who had another complication, such as stroke, myocardial infarction, cancer, or a chronic infectious disease persisted over three months despite appropriate treatment during the 6-month study entry period. After 6 months’ entry period, patients were followed to evaluate new onset of CLI.

The protocol for this retrospective observational study was approved by the Tokushukai Group Institutional Review Board (TGE00830–024) and adhered to the tenets of the Declaration of Helsinki. In terms of informed consent, this report adopted an opt-out consent instead of the written informed consent. We provided patients with information of explaining the proposed research project (the purpose, required individual data and duration of research) by means of an information sheet or a website of the hospital, and gave them the opportunity of opt-out.

### Definition

PAD was defined as a unilateral or bilateral ankle-brachial pressure index of < 0.9. At this cut-off point, HD patients with PAD could be detected with 100% specificity as reported in our previous report [10]. CLI was defined as rest pain or ulcer/gangrene due to limb ischemia. Ankle-brachial pressure index was measured using an ABI-form (Colin, Tokyo, Japan) that simultaneously measured unilateral brachial pressure in the arm without an arteriovenous fistula and ankle blood pressure by using an oscillometric method as described previously [10]. Ankle-brachial pressure index was calculated as the ratio of ankle systolic pressure divided by brachial systolic pressure. CLI was diagnosed if a patient developed ulceration or gangrene in one or both lower extremities due to limb ischemia and required endovascular or surgical intervention.

Dialysis-related hypotension can be divided into orthostatic hypotension, chronic sustained hypotension, and IDH [13]. There is no evidence-based consensus about the definition of IDH, so that IDH prevalence ranged from 15 to 50% of ambulatory HD sessions [14]. We adopted IDH definition according to Flythe et al. [15] and the guideline in the Japanese Society of Dialysis Treatment [13] with modification, that is, a ≥ 20 mmHg decreases in systolic blood pressure compared to 30 min before and a nadir systolic blood pressure < 90 mmHg. Nadir-based definitions best capture the association between IDH and mortality. Although, in the previous study, the 30% frequency threshold was also used to define IDH, this study was so small that we did not adopt this threshold.

### Hemodialysis

Four-hour HD was performed 3 times weekly in all patients. Cellulose triacetate, polysulfone, polyethersulfone, or polyacrylonitrile dialyzer were used, and Kindaly AF-3® (Fuso. Ltd., Tokyo, Japan) was used for dialysate. Qd (dialysate flow) was set at 500 mL/min, and Qb (blood flow) was set at 4–5 mL/min/kg body weight in each patient. Cardio-thoracic ratio on chest X-ray and human atrial natriuretic peptide (hANP) at the end of HD session were evaluated every month, and these parameters were used to adjust dry weight appropriately. Dialysis adequacy was assessed monthly by urea clearance formula (*Kt/V),* where *K* is the blood urea clearance of dialyzer (litter per hour), *t* is dialysis time (hours), and *V* is the distribution volume of urea (litters).

### Data collection

Demographic, clinical, and laboratory data were retrieved from medical records. Demographic and clinical characteristics including age, sex, HD duration, smoking status (both current and former), prevalence of diabetes mellitus and hypertension, history of CVD (including coronary artery disease, heart failure, arrhythmia, and thoracic or abdominal aortic dissection) and PAD were recorded, along with interdialytic weight gain and blood pressure before starting HD. The laboratory data included serum total protein, albumin, calcium, phosphate, glycoalbumin, high-density lipoprotein cholesterol, low-density lipoprotein cholesterol, and hemoglobin. Blood samples were routinely examined before the first HD session at the beginning of the first week of each month between January 2012 and June 2012 at our hospital, and the averages of these data were used as baseline values.

The frequency of IDH was identified from all HD records from January 2012 to June 2012 (78 HD sessions in each patient). We normally measure blood pressure at the beginning and every 30 min until the end of HD session. IDH was defined as ≥20 mmHg decreases in systolic blood pressure compared to 30 min before and a nadir systolic blood pressure < 90 mmHg as mentioned previously.

### Statistical analysis

Continuous variables were expressed as the mean ± standard deviation or as the median and interquartile range. Categorical data were expressed as numbers or proportions. We detected prognostic factors for the outcome using the subdistribution hazards model. To further prove that IDH was a prognostic factor for CLI after adjusting for confounding, a multivariate model was fitted with adjustment for sex, age, smoking and history of CVD. Non-parametric estimates of the risk of CLI incidence were calculated using cumulative incidence function with death as a competing risk. The start of time at risk was from 1st July 2012. The end of time at risk was when patients were revascularized, death or 30th Oct 2014. As for patients lost to follow-up, the exit date was the day when we could follow up. The statistical analyses were conducted using JMP Pro 11 for Windows (SAS Institute, Tokyo, Japan) and using the R version 3.5.2 (R Foundation for Statistical Computing, Vienna, Austria) for cumulative incidence function.

## Results

### Baseline characteristics and outcome

**Fig. 2:**
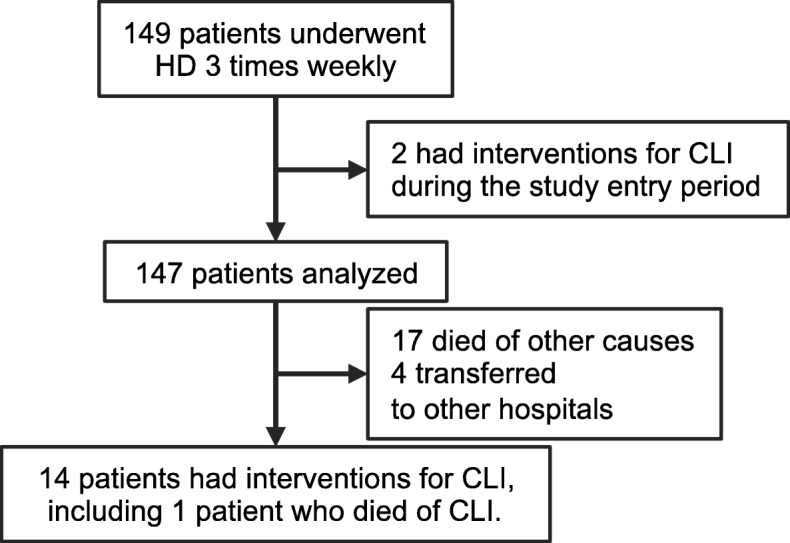
Flow of study participants. Abbreviation: CLI, critical limb ischemia; HD, hemodialysis

In total, 147 of the 149 patients who received HD on an outpatient basis at our hospital during the study period were eligible for inclusion (Fig. 2). The baseline patient characteristics are shown in Table 1. The mean patient age was 70.9 years and the median HD duration was 112 months. The proportions of female patients and patients with diabetes mellitus were 38.6 and 42.9%, respectively. Mean follow-up duration was 24.5 months.

**Table 1:** Patient characteristics at baseline

	All (*N* = 147)	IDH (−) (*N* = 97)	IDH (+) (*N* = 50)
Age (y)	70.9 ± 11.1	71.1 ± 11.0	70.6 ± 11.4
Male/female	90/57	61/36	29/21
Time since starting HD (months)	112 (68–167)	121 (74–173)	108 (60–157)
Diabetes mellitus	63 (42.9)	35 (36.1)	28 (56.0)
Hypertension	131 (89.1)	84 (86.6)	47 (94.0)
Smoking	60 (40.8)	40 (41.2)	20 (40.0)
History			
CVD (%)	55 (37.4)	31 (32.0)	24 (48.0)
PAD (%)	16 (10.9)	6 (6.1)	10 (20.0)
Medication			
Antiplatelet agents (%)	88 (59.8)	57 (58.8)	31 (62.0)
ARB (%)	95 (64.6)	61 (62.9)	34 (68.0)
ACEI (%)	7 (4.8)	5 (5.2)	2 (4.0)
CCB (%)	83 (56.5)	59 (60.8)	24 (48.0)
β-blocker (%)	44 (29.9)	31 (32.0)	13 (26.0)
Characteristics on dialysis			
IDWG (%)	3.91 ± 1.39	3.74 ± 1.36	4.25 ± 1.41
Systolic BP (mmHg)	142.3 ± 19.9	141.6 ± 18.3	143.6 ± 23.0
Diastolic BP (mmHg)	74.7 ± 13.2	75.6 ± 12.1	72.9 ± 15.3
Blood flow rate (mL/min)	213 ± 33	215 ± 33	208 ± 32.8
Kt/V urea	1.51 ± 0.26	1.50 ± 0.27	1.52 ± 0.25
Dialyzer: PS/PES	105 (71.4%)	74 (76.3)	31 (62.0)
Dialyzer: cellulose triacetate	35 (23.8%)	18 (18.6)	17 (34.0)
IDH	50 (34.5%)	0 (0%)	50 (100%)
Laboratory data			
Albumin (g/dL)	3.68 ± 0.29	3.72 ± 0.24	3.58 ± 0.36
Corrected Calcium (mg/dL)	8.33 ± 0.68	8.32 ± 0.68	8.35 ± 0.70
Phosphate (mg/dL)	5.28 ± 1.02	5.20 ± 0.96	5.44 ± 1.13
Intact PTH (pg/mL)	204.3 ± 126.9	201.2 ± 119.7	210.4 ± 141.0
HDL-C (mg/dL)	48.5 ± 13.8	49.5 ± 14.5	46.6 ± 12.4
LDL-C (mg/dL)	78.3 ± 23.2	81.0 ± 23.2	73.3 ± 22.8
Glycoalbumin (%)	17.9 ± 4.7	17.5 ± 4.4	18.9 ± 5.2
Hemoglobin (g/dL)	11.1 ± 0.79	11.1 ± 0.82	11.2 ± 0.72
β2-microglobulin (mg/L)	27.2 ± 5.10	26.8 ± 5.15	28.0 ± 4.95

The data are presented as the mean ± standard deviation, median (interquartile range), or number (percentage). Abbreviations: *ARB* angiotensin receptor blocker; *ACEI* angiotensin-converting enzyme inhibitor; *BP* blood pressure; *CCB* calcium channel blocker; *CVD* cardiovascular disease; *HD* hemodialysis; *HDL-C* high-density lipoprotein cholesterol; *IDH* intradialytic hypotension; *IDWG* interdialytic weight gain; *LDL-C* low-density lipoprotein cholesterol; *PAD* peripheral arterial disease; *PES* polyethersulfone; *PS* polysulfone; *PTH* parathyroid hormone

In 72 sessions of HD in the study entry period, 50 patients had episodes of IDH and 97 patients did not show IDH. The frequency of IDH distributed from once to 24 times. However, 80% of patients showed IDH less than 6 times.

**Fig. 3:**
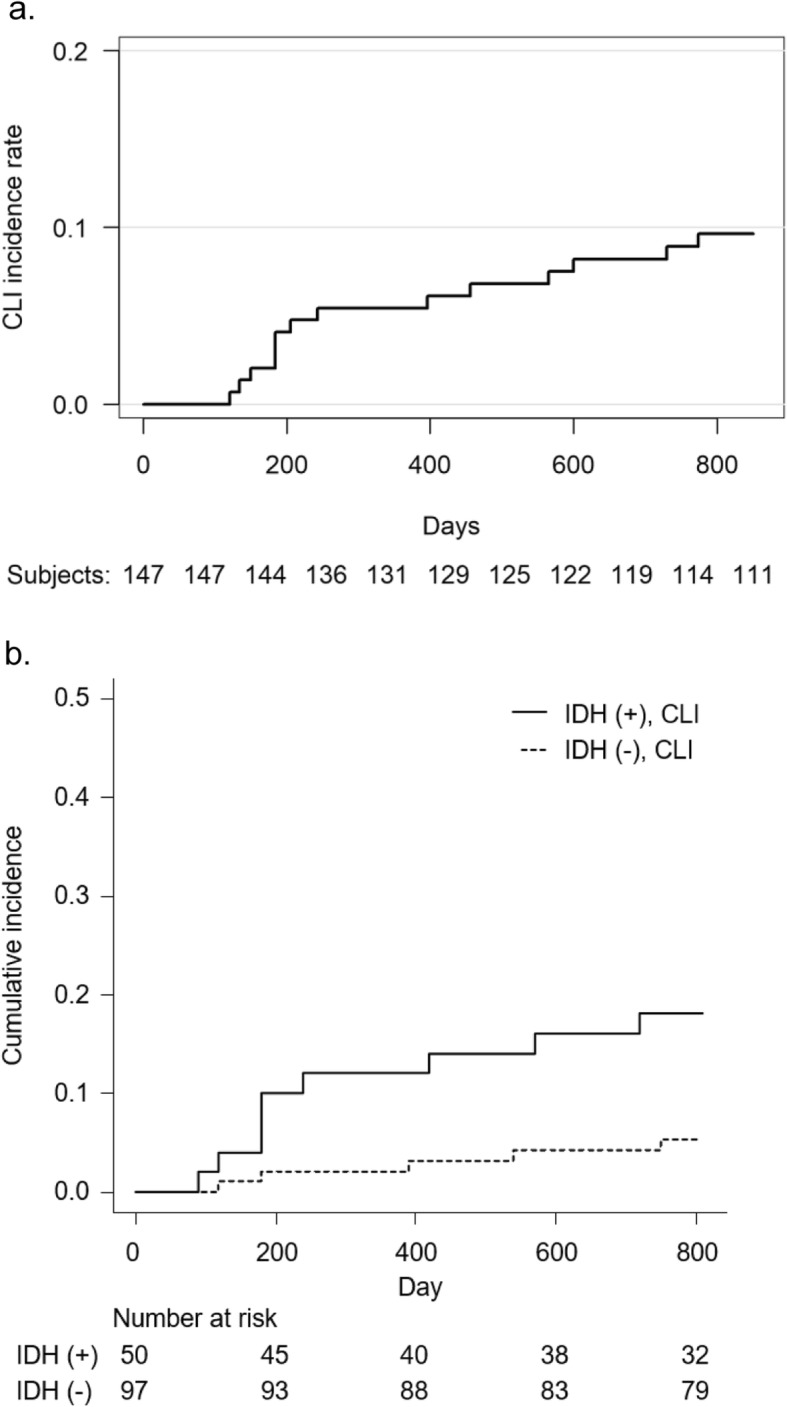
**a** Incidence of intervention for or death from CLI. Abbreviation: CLI, critical limb ischemia. **b** The estimation of the cumulative incidence function of CLI by history of IDH with death as a competing event. CLI, *P* = 0.01: Gray’s test. Abbreviations: CLI, critical limb ischemia; IDH, intradialytic hypotension. The number at risk of IDH(+) and IDH(−) groups are shown under the estimation of the cumulative icidence function of CLI

During follow-up, 14 patients underwent interventions for incident CLI. Among these patients, 13 patients had already been diagnosed as having PAD, and IDH was found in 9 out of 14 patients. Endovascular therapy was performed in all these patients (Fig. 2). Cumulative incidence function estimates revealed 1-year and 2-year incident CLI rates of 5.5 and 9.6%, respectively (Fig. 3a). One patient died of CLI, and 17 patients died of non-CLI causes during the follow up period, i.e., stroke in 4, pneumonia in 4, cancer in 4, severe arrhythmia in 2, dissociation of thoracic aorta in 1, aortic valve stenosis in 1, and sepsis in 1. Four patients were lost to follow-up because of transfer to other hospitals (Fig. 2).

### Predictors for CLI

The variables subjected to the subdistribution hazards model are listed in Table 2. Factors associated with incident CLI in univariate analysis were age, smoking, diabetes mellitus, PAD, history of CVD, and IDH (Table 2). In a multivariate model, IDH was also significantly associated with CLI with subdistribution hazard ratio of 3.13 (1.05–9.37) (Table 3).

**Table 2:** Univariate subdistribution hazard ratios for predictors of intervention for or death from critical limb ischemia

		95% Confidence Interval		*p*-value
	SHR	Lower	Upper	
Age^a^	1.79	1.21	2.84	0.002
Male	0.85	0.25	2.84	0.79
Time since starting HD^b^	1.00	0.997	1.00	0.83
Smoking^c^	2.78	0.95	8.2	0.06
Diabetes Mellitus	8.74	2.0	38.2	0.004
PAD	4.95	1.72	14.2	0.003
History of CVD	4.51	1.44	14.1	0.01
Serum albumin^d^	0.36	0.08	1.56	0.17
Serum calcium^e^	0.91	0.44	1.87	0.8
Serum phosphate^e^	1.16	0.73	1.85	0.54
Serum LDL-C^f^	0.90	0.73	1.10	0.31
Serum intact PTH^g^	1.00	0.99	1.00	1.00
Serum β2-microglobulin ^h^	1.05	0.96	1.15	0.3
Hemoglobin^d^	0.89	0.51	1.54	0.67
IDH^i^	3.73	1.26	11.0	0.02
IDWG^j^	1.18	0.80	1.75	0.40
Kt/V urea^k^	1.47	0.33	6.56	0.62

^a^hazard ratio per 1 year; ^b^hazard ratio per 12 months; ^c^includes current smokers and ex-smokers; ^d^hazard ratio per 1.0 g/dL; ^e^hazard ratio per 1.0 mg/dL; ^f^hazard ratio per 10 mg/dL; ^g^hazard ratio per 1 pg/mL; ^h^hazard ratio per 1.0 mg/L; ^i^categorical variables; ^j^hazard ratio per 1%; ^k^hazard ratio per 1. The reference category was the group without IDH during the study period. Abbreviations: *CVD* cardiovascular disease; *IDH* intradialytic hypotension; *IDWG* interdialytic weight gain; *LDL-C* low-density lipoprotein cholesterol; *PAD* peripheral arterial disease; *PTH* parathyroid hormone; *SHR* subdistribution hazard ratio

**Table 3:** Multivariate subdistribution hazards ratios for predictors of intervention for or death from critical limb ischemia

	SHR (95% CI)	*p*-value
Age^a^	1.48 (0.85–2.37)	0.18
Male	2.26 (0.60–8.55)	0.23
Smoking^b^	5.48 (1.47–20.4)	0.01
History of CVD	3.26 (0.91–11.7)	0.07
IDH^c^	3.13 (1.05–9.37)	0.04

^a^Hazard ratio per 10 years; ^b^includes current smokers and ex-smokers; ^c^categorical variables. The reference category was the group without IDH during the study period. Abbreviations: *CI* confidence interval; *CVD* cardiovascular disease; *HR* hazard ratio; *IDH* intradialytic hypotension; *PAD* peripheral arterial disease; *SHR* subdistribution hazard ratio

The estimation of cumulative incidence function of CLI with death as a competing event demonstrated that the group with IDH (IDH(+)) during the study entry period had significantly more interventions and death attributable to CLI than the group without IDH (IDH(−)) (CLI, *P* = 0.01: Gray’s test; Fig. 3b). In the IDH(+) group, the 1-year and 2-year incident CLI rates were 12.0 and 18.1%, respectively. The new onset of CLI was significantly higher in the IDH(+) group than in the IDH(−) group.

## Discussion

Our study demonstrated that 1-year and 2-year overall incidence of new onset CLI among patients on maintenance HD was 5.5 and 9.6%, respectively, and IDH was significantly and independently associated with incident CLI. In IDH(+) group, 1-year and 2- year frequency of new onset CLI was 12.0 and 18.1%. To our knowledge, this is the first report showing IDH to be a significant predictor of CLI in patients on HD.

CLI is a devastating condition that leads to infections, major amputations, and death in patients on HD [2, 16, 17]. Appropriate management of risk factors for CLI is needed to improve survival in these patients. The risk factors for PAD are known to include older age, male sex, smoking, hypertension, and diabetes mellitus [9]. However, the majority of patients on HD already have several risk factors for PAD, typically older age, hypertension, diabetes mellitus, and history of atherosclerotic disease. Therefore, different approaches are needed to prevent CLI in these patients. In this regard, the evidence that IDH contributes to CLI is clinically significant.

Although the precise mechanism of association between IDH and CLI is not known, there might be an association between IDH and vascular defects in the lower limbs. The pathophysiology of CLI involves reduced perfusion and a hypoxic state in the area of injury. The distal arterioles adapt to chronic ischemia by a decrease in wall thickness and vasodilatation, which leads to an increase in distal hydrostatic pressure and distal edema. Concomitant inflammation, when present, induces endothelial dysfunction, which in turn leads to more microthrombosis and edema in the lower extremities [18]. When IDH occurs, the lowered blood pressure would not be able to deliver enough perfusion to the stenotic arteries and injured tissue [19]. A short-term hypoxic state would exacerbate the progression of CLI. Vascular calcification is also likely to be involved in the pathophysiology described above [20].

IDH is a common event during HD. In one study, at least 10% of HD patients had IDH episodes (defined as systolic blood pressure < 90 mmHg) [15]. In our study, 34.5% of patients had IDH episodes during the 6-month study period. Although the mechanism by which IDH develops is unclear, there have been reports suggesting that a lower dry weight and a high ultrafiltration rate are associated with the frequency of IDH and that an increase in dry weight decreases the number of episodes of IDH [21–23]. Therefore, to prevent progression of CLI, it might be best to avoid setting an excessively low dry weight to control blood pressure. Our study also found that smoking was a risk factor for CLI in patients on HD, as it is in the general population. However, it is better to mention that there is a limitation of this analysis because almost all smokers were male and the hazard ratio might seem to be biased. If our study had many women’s smokers, the result might be different.

This study had some limitations. It was a retrospective single-center study with small sample size. Therefore, IDH and other factors such as diabetes and hypertension could be still confounding. A further prospective multicenter trial with larger cohorts is needed to confirm that IDH is independently associated with CLI. Additionally, the possibility that IDH was a result of cardiac dysfunction or impaired autonomic function caused by diabetes mellitus cannot be ruled out. However, as mentioned earlier, this possibility seems unlikely given that none of our patients underwent procedures for impaired cardiac function during the study period. IDH was found to be an independent predictor of the incidence of CLI in our analysis. A further interventional study is necessary to determine whether preventing IDH will decrease the risk of CLI in patients on HD. Careful consideration should be also taken into the population in this study, which consists of older patients with longer HD vintage than the hemodialysis population. However, we think that the Japanese HD population consists in older patients with longer HD vintage than those in other countries [24, 25], and that this population in our study was the representative of the Japanese HD population. Moreover, clinical practice in Japan, which may be different from that in other countries, might have some effect on the result. For example, the guideline in Japanese Society for Dialysis Treatment recommends the lower target of PTH level under 240 pg/mL [26] and this cohort had the median PTH of 200 pg/mL. Given that the association with PTH and vascular calcification, the possibility would raise that the PTH management may have an impact on the total number of the outcome. However, univariate Cox regression model revealed no association with PTH and CLI, which suggests that PTH has little impact on the incidence of CLI.

## Conclusion

IDH was an independent predictor for incident CLI in patients on HD. Further studies are now required to find ways of improving management of patients on HD to avoid IDH and decrease the risk of CLI.

## Data Availability

The datasets used and/or analyzed during the current study available from the corresponding author on reasonable request.

## Abbreviations

CLI: Critical limb ischemia
CVD: Cardiovascular disease
HD: Hemodialysis
HDL-C: High-density lipoprotein cholesterol
IDH: Intradialytic hypotension
IDWG: Interdialytic weight gain
LDL-C: Low-density lipoprotein cholesterol
PAD: Peripheral artery disease
PTH: Parathyroid hormone
